# Contemporary Management of Cerebrospinal Fluid Rhinorrhoea: A Review of the Literature

**DOI:** 10.3390/jcm14030995

**Published:** 2025-02-04

**Authors:** Zahir Mughal, Pablo Martinez-Devesa, Alexandros Boukas, Sanjeeva Jeyaretna, Ali Qureishi

**Affiliations:** 1 Department of ENT, John Radcliffe Hospital, Headley Way, Headington, Oxford OX3 9DU, UK; pablo.martinez-devesa@ouh.nhs.uk (P.M.-D.); ali.qureishi@ouh.nhs.uk (A.Q.); 2 Department of Neurosurgery, John Radcliffe Hospital, Headley Way, Headington, Oxford OX3 9DU, UK; alex.boukas@ouh.nhs.uk (A.B.); sanjeeva.jeyaretna@ouh.nhs.uk (S.J.); 3 Nuffield Department of Clinical Neurosciences, University of Oxford, John Radcliffe Hospital, Headley Way, Headington, Oxford OX3 9DU, UK

**Keywords:** anterior skull base, cerebrospinal fluid, endoscopic, idiopathic intracranial hypertension, rhinorrhoea

## Abstract

**Background/Objectives:** Cerebrospinal fluid (CSF) rhinorrhoea carries a significant risk of life-threatening intracranial complications. This review provides a contemporary overview of current management strategies for CSF rhinorrhoea. **Methods:** We conducted a literature review, examining studies from Medline, Embase, and Google Scholar published within the last 20 years. This narrative synthesis summarises the current and future trends in the management of CSF rhinorrhoea. **Results:** The management of CSF leaks requires a multidisciplinary approach, encompassing a thorough clinical assessment, targeted diagnostic testing, and a spectrum of surgical and non-surgical interventions. Endoscopic techniques, particularly the use of vascularised flaps such as the nasoseptal flap, has become central to anterior skull base reconstruction. Numerous graft and flap choices provide tailored solutions based on defect size and CSF flow characteristics, with reported success rates exceeding 90%. **Conclusions:** Endoscopic repair of CSF rhinorrhoea continues to evolve, with modern techniques significantly enhancing success rates and reducing morbidity. Further understanding of underlying aetiologies, advances in technology, and refinement in surgical technique are areas for future innovation in CSF rhinorrhoea management.

## 1. Introduction

Cerebrospinal fluid (CSF) is primarily produced by the choroid plexus within the ventricles at an approximate rate of 0.35 mL/min, equating to 350–500 mL/day [1]. It circulates through the ventricular system and subarachnoid space, where it serves a role in cushioning the brain, regulating intracranial pressure, and clearing metabolic waste. Reabsorption occurs into the venous system via arachnoid villi. In adults, the total CSF volume averages 140 mL, with the total volume being turned over approximately three times per day [1]. CSF rhinorrhoea is characterised by abnormal communication between the subarachnoid space and the nasal cavity. This occurs due to a defect in the dura mater, skull base bone, and nasal mucosa, creating a fistulous connection.

**Figure 1:**
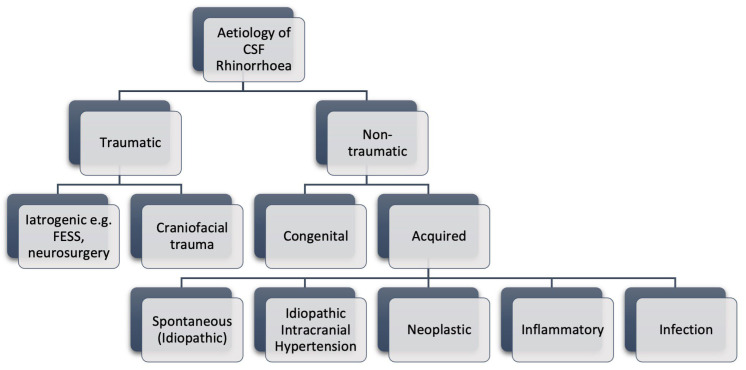
Classification of the aetiology of CSF rhinorrhoea. FESS = Functional Endoscopic Sinus Surgery.

The aetiology of CSF rhinorrhoea can be broadly classified into traumatic or non-traumatic (Figure 1), with craniofacial trauma being the most common cause [1]. Anterior skull base fractures account for nearly 25% of all skull fractures, emphasising their clinical relevance [2]. A systematic review identified the most common sites of injury as the ethmoid/cribriform plate (46%), frontal sinus (44%), orbital roof (15%), and sphenoid sinus (11%) [2]. The incidence of spontaneous CSF leaks is rising, particularly amongst females in regions with high obesity rates [3]. Traditionally, spontaneous CSF leaks have been defined as occurring without an identifiable inciting factor. However, growing evidence suggests that a substantial proportion of these cases are attributable to underlying idiopathic intracranial hypertension (IIH) [4]. IIH predominantly affects middle-aged obese females and is characterised by symptoms and signs of raised intracranial pressure (ICP) in the absence of hydrocephalus or mass lesions, a normal CSF composition, and no underlying cause for the elevated ICP [5]. Epidemiological data indicate a correlation between the rising levels of obesity and the increasing incidence of spontaneous CSF leaks [6]. Since obesity is a known risk factor for IIH, this association reinforces the hypothesis that many spontaneous leaks are secondary to IIH. The proposed mechanism for CSF leakage in IIH involves elevated ICP and associated dural pulsations, which can exert direct pressure on the skull base, leading to bone thinning, resorption, and formation of meningoceles or meningoencephaloceles [7]. These defects are most commonly observed in the sphenoid sinus (30%), ethmoid sinus (17%), and cribriform plate (16%) [8].

CSF rhinorrhoea carries a significant risk of life-threatening intracranial complications, including meningitis, seizure, pneumocephalus, epidural abscess, and hydrocephalus [9]. Prompt diagnosis and treatment are therefore essential to prevent these complications. The advent of endonasal endoscopic techniques has greatly expanded the armamentarium of reconstructive methods for anterior skull base defects. This review aims to provide an updated overview of the contemporary assessment and management of CSF leaks originating from the anterior skull base. We highlight current innovations in treatments and future potential directions.

## 2. Materials and Methods

A literature review was conducted by searching the Medline, Embase, and Google Scholar databases using the following key terms: CSF, cerebrospinal fluid, leak, rhinorrh*, fistula, defect, endoscop*, repair, closure, reconstruction, management, treatment, approach, and seal*. Additional studies were identified through forward citation tracking and manually reviewing reference lists. Articles published in the last 20 years, focusing on diagnosis, management, and surgical techniques for anterior skull base defects, were included. We excluded studies focusing on indirect CSF rhinorrhoea from the middle or posterior fossae via the eustachian tube. Articles were selected based on their relevance, with the aim of identifying key themes and discussing recent advancements in the field of CSF rhinorrhoea. The analysis was narrative in nature, synthesising findings qualitatively.

## 3. Results

### 3.1. Clinical Assessment

The clinical presentation of CSF rhinorrhoea is typically unilateral, clear, watery nasal discharge [10]. The clinical history should focus on the consistency of the nasal discharge, laterality, duration, onset, timing, aggravating factors, such as straining or posture, and any episodes of meningitis. Symptoms of raised intracranial pressure, such as headaches associated with visual disturbance, should be elicited. However, patients with an active CSF leak may paradoxically present with symptoms of reduced ICP, including orthostatic headache and neck stiffness [10]. The history should also explore potential underlying causes, such as intracranial pathology, prior head trauma, or sinonasal surgery. Particular attention should be paid to identifying IIH, a condition that can result in significant morbidity, including permanent visual loss and disabling headache [11]. IIH is typically seen in females of childbearing age and is associated with obesity, obstructive sleep apnea, and hypertension [3]. Symptoms include headache, visual obscuration, blurry vision, diplopia, pulsatile tinnitus, dizziness, and neck pain [12]. These symptoms may be masked by the presence of a CSF leak, which can act as a ‘pressure release valve’ [13]. Consequently, symptoms of IIH may only manifest after the leak is repaired, complicating the preoperative diagnosis of this condition.

A comprehensive ENT examination is essential, with particular emphasis on nasendoscopy for direct visualisation of the leak. This evaluation should also include the inspection of the Eustachian tubes and an otologic examination to identify otologic sources of CSF rhinorrhoea [10]. The ‘halo sign’ [8], characterised by an internal blood ring and an external lighter fluid ring on filter paper, may indicate the presence of CSF in traumatic cases. Subtle CSF leaks may be provoked by having the patient sit up, lean forward, and flex the neck after lying supine—known as the ‘reservoir sign’ [14]. The remainder of the examination focuses on neuro-ophthalmology signs.

### 3.2. Investigations

Confirmation of CSF rhinorrhoea requires laboratory analysis of nasal fluid for beta-2 transferrin or beta-trace protein [8,15]. Once CSF has been confirmed, imaging studies are essential to localise the defect. High-resolution computed tomography (HRCT) of the skull base and paranasal sinuses is the initial imaging of choice, providing detailed bony anatomy [15]. If the osseous defect is not visualised on CT, magnetic resonance imaging (MRI) with heavily T2-weighted sequences and fat suppression is recommended for detecting CSF fistula [16]. The MRI protocol should utilise fast imaging employing steady-state acquisition cycled phases (FIESTA-C) or constructive interference steady state (CISS), combined with fluid attenuation inversion recovery (FLAIR) sequences, or their equivalents [10]. At our institution, we prefer a dual imaging approach incorporating both CT and MRI, leveraging their complementary strengths to improve the localisation of CSF leak(s) and facilitate precise surgical planning. This approach is particularly beneficial for patients with spontaneous CSF rhinorrhoea or IIH, who often present with multiple skull base defects [10].

For cases with active leaks that remain undetected by non-invasive imaging, CT cisternography with intrathecal dye injection may be more definitive [15]. Additionally, intrathecal fluorescein (ITF) administration may aid in intraoperative identification of the fistula [17]. However, this off-label use of fluorescein carries rare but potentially serious neurological complications [17]. The use of intraoperative neuronavigation with CT (with or without MRI fusion) is recommended in complex cases and can assist in localising the area of bony defect and associated CSF leak [10].

The diagnostic workup for suspected IIH follows recommendations of recent international consensus guidelines [10,12]. It begins with an ophthalmology evaluation to assess for papilledema and visual function. If papilledema is detected, the first step is to measure blood pressure to exclude malignant hypertension, followed by an urgent MRI venogram. Radiological indicators of IIH include an empty sella, posterior globe flattening, tortuosity of the optic nerve, optic nerve sheath distention, and widening of the foramen ovale [18]. Transverse sinus stenosis is considered to be the most sensitive and reliable imaging indicator marker for IIH [19]. After ruling out hydrocephalus, mass, structural, vascular lesion, and abnormal meningeal enhancement on imaging, all patients should have a lumbar puncture. This test is used to ensure that the composition is normal and to measure opening pressures, with >25 cm of CSF consistent with a diagnosis of IIH [10]. This diagnostic approach aligns with the modified Friedman criteria [20].

### 3.3. Management

Management of CSF leaks is multidisciplinary, typically including skull base surgeons (ENT and neurosurgery), ophthalmologists, neurologists, and radiologists, coordinated through a skull base multidisciplinary team meeting.

**Figure 2:**
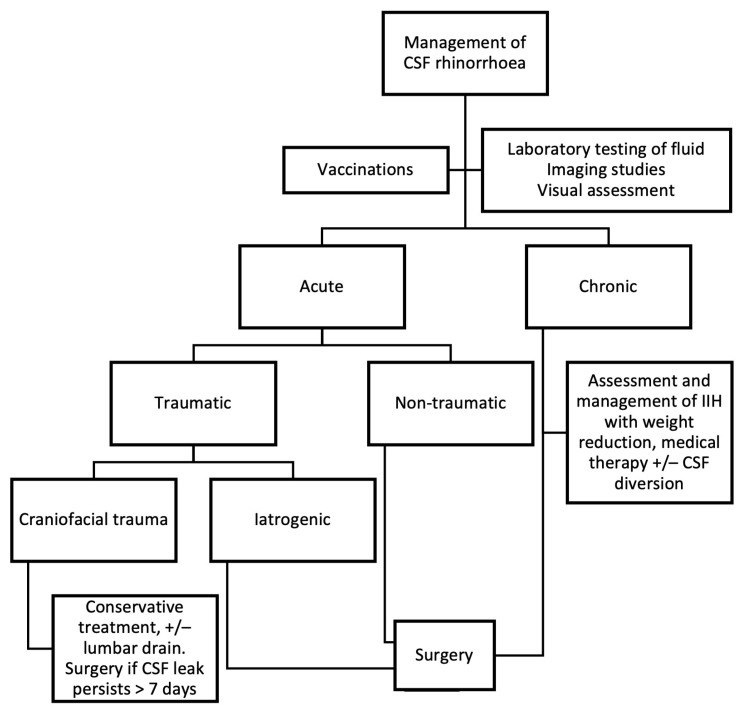
Algorithm for the management of cerebrospinal fluid (CSF) rhinorrhoea. IIH—Idiopathic intracranial hypertension.

Once the diagnosis has been established, vaccination should be administered against common pathogens (*Streptococcus pneumoniae*, *Haemophilus influenzae* type b, and *Neisseria meningitidis*) to prevent ascending bacterial meningitis [21]. The approach to management depends on whether the leak is acute or chronic [1]. The management algorithm is summarised in Figure 2.

In acute traumatic CSF leaks, management varies based on the cause. Iatrogenic surgical trauma is unlikely to resolve spontaneously and therefore requires prompt surgical treatment [15]. In contrast, leaks from accidental craniofacial trauma are likely to resolve spontaneously and can often be initially managed conservatively with bed rest, head elevation by 30 degrees, avoidance of straining, and laxatives [1]. Lumbar drains may reduce the duration of leakage during conservative management [15]. A step-wise escalation has been proposed, with a three-day trial of conservative treatment, followed by three days of CSF diversion [22], and surgical repair is considered if the leak persists beyond seven days despite these measures [15]. Prophylactic antibiotics are generally not recommended for this group, as they do not significantly reduce infection risk [23].

For delayed presentations of traumatic CSF rhinorrhoea, and all non-traumatic cases, the mainstay of management is surgical repair. Since the introduction of rigid endoscopes for transnasal skull base repair in 1981 [24], the endoscopic approach has become the standard of care, achieving success rates exceeding 90% [25]. This minimally invasive technique has largely replaced traditional open craniotomy approaches due to reduced morbidity and mortality [26].

### 3.4. Surgical Technique

**Figure 3:**
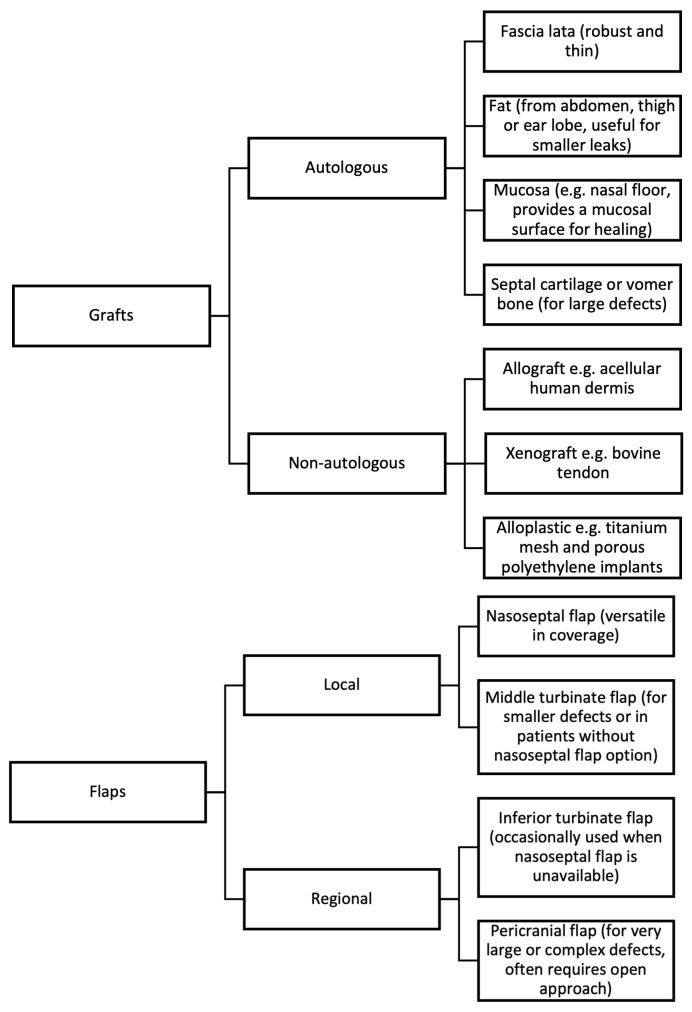
Common materials used in anterior skull base reconstruction.

The surgical technique for endoscopic repair involves several key steps [10,22]: the mucosa surrounding the defect is meticulously removed circumferentially to expose the underlying bone. If a meningocele or meningoencephalocele is present, it is reduced using bipolar diathermy and resected to the level of the skull base. Careful inspection is necessary to identify and protect any herniated cerebral vessels, which must be placed back into the intracranial space to avoid a distal infarct. The defect is reconstructed using a variety of grafts and flaps, as outlined in Figure 3. Autologous materials are commonly used but require a donor site, introducing additional risks, such as infection, hematoma, or seroma formation, and a longer operation time [27]. Since the description of the vascularised nasoseptal flap in 2006 [28], this flap has become the workhorse for anterior skull base reconstruction. While allografts and xenografts present an alternative, mitigating donor site morbidity, they carry risks of foreign body reaction and aseptic meningitis [14]. Alloplastic materials provide another option, but they remain susceptible to complications such as infection and extrusion [14,27]. Currently, there is no definite evidence favoring one type of material over another, and material selection is typically based on the surgeon’s preference [29].

Grafts can be placed as under-lay, on-lay, or in combination. Techniques such as ‘bath-plug’ [30] or ‘parachute’ [31] involve intradural placement of grafts to achieve a secure seal. For small defects, a single-layer closure with a graft may be sufficient, while multi-layered reconstruction with grafts and vascularised flaps is preferred for large or high-flow leaks [22,29]. For larger osseous defects or high-flow leaks, the gasket-seal technique [32] can be employed, incorporating a rigid buttress of bone or cartilage or Medpor (alternative synthetic material) between the layers to enhance structural support. Some authors advocate for over-correction, even in smaller defects, to accommodate for an expected 20% shrinkage in mucosal tissue postoperatively [8,33]. Stabilisation of the reconstruction is achieved with various adjunctive materials, including glues, sealants, and nasal packing. While these adjuncts are commonly used, evidence supporting their effectiveness is limited [29]. Our usual practice involves a multi-layered reconstruction with autologous grafts and nasoseptal flap, and lumbar drainage is generally avoided unless strongly indicated, such as in cases of high-flow leaks or revision surgeries.

### 3.5. Postoperative Care

Postoperative management includes maintaining head elevation at 30 degrees, bed rest, laxatives, and avoidance of straining for six weeks [22]. Patients should be admitted overnight and undergo monitoring of vital and neurological signs [10]. A temporary lumbar drain may be beneficial in cases of raised intracranial pressure (ICP) [29]. An early postoperative CT scan is performed to check graft position and exclude postoperative intracranial complications such as pneumocephalus, which may predict repair failure [34]. If the scan is satisfactory, patients are typically allowed to mobilise after 48 h, although evidence supporting the optimal duration of bed rest remains limited. Upon discharge, patients are instructed to use a saline nasal spray for six weeks [22]. As dissection occurs through a clean–contaminated operative field, most surgeons elect to use perioperative antibiotics with anti-staphylococcal coverage [14]. At ENT follow-up review, the nasal cavity is assessed for mucosal healing and identification of postoperative problems such as sinonasal infection, CSF leak recurrence, and intracranial complications.

Patients with spontaneous CSF leaks require close neurology and ophthalmological follow-up to monitor for papilledema and to formally assess intracranial pressure, which may develop after closure of the skull base defect if they have underlying IIH [10,13].

### 3.6. Idiopathic Intracranial Hypertension

**Figure 4:**
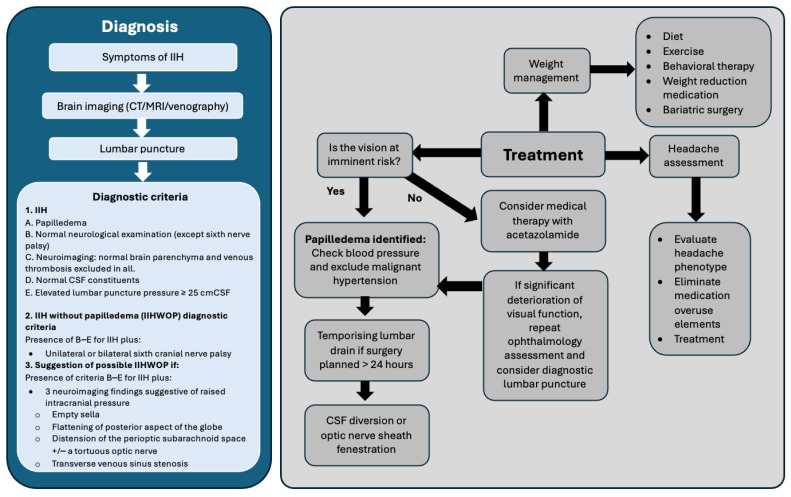
Algorithm for the management of idiopathic intracranial hypertension. Adapted from Mollan et al. [12].

The management of IIH involves collaboration between ENT surgeons, neurologists, ophthalmologists, and neurosurgeons. The three primary goals of IIH treatment are to reduce body weight, aid vision protection, and headache control [35]. The management of IIH is summarised in Figure 4. Weight reduction is the only disease-modifying therapy currently available for IIH and is recommended for all patients with a body mass index (BMI) greater than 30 kg/m^2^ [11]. A systematic review of weight loss strategies demonstrated that lifestyle interventions achieved modest weight reductions ranging from 1.4 to 15.7 kg, while bariatric surgery yielded significantly greater weight loss, with an average reduction of 27.2–27.8 kg at 24 months [36]. Weight loss has been shown to correlate with a decrease in ICP, with bariatric surgery producing the most pronounced reduction, averaging 11.9 cm H_2_O at 24 months [36]. This highlights the importance of weight management in contributing to IIH disease remission. In view of this, expert consensus recommends considering bariatric surgery referral for patients with a BMI ≥ 35 kg/m^2^ [36].

Acetazolamide, a carbonic anhydrase inhibitor that interferes with CSF production, is considered the mainstay of pharmacological treatment of IIH [11], although evidence supporting its efficacy is not strong [37]. Additionally, due to its side-effect profile, it is not routinely prescribed in the UK [12]. Alternative medications that have been used in the management of IIH include furosemide, topiramate, and octreotide [38]. If medical therapy fails, is poorly tolerated, or if vision is threatened, surgical intervention such as CSF diversion procedures or optic nerve sheath fenestration (ONSF) may be necessary, with the choice of procedure depending on local expertise [11,12]. In urgent sight-threatening cases, a temporising lumbar drain may serve as a bridge to surgery, providing protection for vision [12].

Promising upcoming medical therapies include 11β-HSD1 inhibitors, glucagon-like peptide-1 receptor agonists (GLP-1RAs), anti-calcitonin gene-related peptide (CGRP), and receptor monoclonal antibodies [11,35,38]. Cerebral venous sinus stenting (VSS) has also gained attention as an emerging surgical intervention, demonstrating significant improvements in papilledema, headache, and pulsatile tinnitus rates, with a superior safety profile compared to CSF shunting and ONSF [38]. These novel approaches hold promise for improving outcomes and expanding future treatment options in IIH management.

## 4. Discussion

### 4.1. Summary of Main Findings

CSF rhinorrhoea presents significant challenges in both diagnosis and management due to its diverse clinical presentations, aetiologies, and treatment approaches. This review highlights the importance of a systematic and multidisciplinary approach to clinical assessment, advanced imaging, pharmacological therapies, and tailored treatments. IIH adds further complexity to the diagnostic process during an active CSF leak, as elevated opening pressures may not be demonstrable, necessitating a high index of clinical suspicion. Beyond repairing the CSF leak, effective management of IIH requires a comprehensive strategy involving weight reduction, pharmacotherapy, and surgical interventions. This holistic approach is essential to address the underlying condition and prevent recurrence or long-term complications.

This review highlights the significant advancements in imaging and surgical techniques for the management of CSF rhinorrhoea, which have evolved considerably over the past two decades. Endoscopic repair has become the standard of care, offering a minimally invasive approach with excellent outcomes. Among these advancements, the use of the vascularised nasoseptal flap has shown remarkable effectiveness in achieving durable closure, especially for high-flow or complex skull base defects. While conservative management remains a viable option for acute, non-iatrogenic traumatic leaks, often with good outcomes, non-resolving or chronic leaks typically require surgical intervention.

### 4.2. Implications of Findings

The findings carry key implications for clinical practice. First, the importance of a multidisciplinary approach, as effective management requires collaboration among ENT surgeons, neurosurgeons, ophthalmologists, radiologists, and neurologists. Ideally, this collaboration should occur within a dedicated skull base MDT to ensure coordinated care. Second, the results highlight the need for early and accurate diagnosis of CSF rhinorrhoea with laboratory testing and precise localisation with advanced skull base imaging techniques. Third, while the adoption of endoscopic techniques has transformed surgical management, the need for individualised treatment based on aetiology, defect size, and CSF leak flow characteristics is central to effective management. Lastly, the recognition and management of underlying IIH in spontaneous cases of CSF rhinorrhoea is essential to prevent recurrence and associated morbidity.

### 4.3. Limitations

This review is limited by challenges within the existing body of literature. Many recommendations are derived from retrospective, observational studies and expert consensus, which may limit their generalisability and strength of evidence. There is a paucity of randomised controlled trials evaluating optimal surgical repair strategies. The evidence supporting pharmacological strategies for ICP control and headache management in IIH remains insufficient, highlighting the need for more robust, high-quality studies in these areas.

As a narrative review, this study did not include the systematic data extraction or analytic techniques characteristic of systematic reviews. However, it provided a broad and comprehensive exploration of the available evidence, identifying trends and highlighting areas for future research.

### 4.4. Future Research

Future research should focus on high-quality, prospective studies and randomised controlled trials to address the current gaps in evidence for the diagnosis and management of CSF leaks. Habenbacher et al. [39] proposed a diagnostic protocol combining beta trace protein testing, HRCT, and ITF for reliably confirming and localising CSF leaks. ITF was instrumental in identifying multiple defects and ensuring watertight closure during surgical repair. However, their study highlighted limitations, such as the potential for false negatives with ITF and cases where leaks were not confirmed intraoperatively despite positive beta trace and HRCT findings. These observations underscore the need for further refinement of diagnostic protocols. Advancements in technology, including MRI sequences, angled scopes, navigation systems, and high-definition cameras [40], could potentially improve the localisation of small or complex leaks and potentially reduce the need for invasive procedures like ITF. Additionally, these advances may facilitate further innovation in endoscopic techniques and reconstructive options.

A diagnosis of IIH is often made retrospectively, as it can be challenging to meet the diagnostic criterion of elevated opening lumbar pressures during an active leak. Future research should aim to develop and validate diagnostic approaches, including the use of characteristic radiological findings, to identify these patients more reliably.

## 5. Conclusions

CSF rhinorrhoea from anterior skull base defects poses a significant clinical challenge. The advent of endoscopic techniques and vascularised flaps, such as the nasoseptal flap, have revolutionised the management of these leaks, offering effective and less invasive solutions with high success rates. A multidisciplinary approach is essential, with tailored interventions based on the nature of the defect and underlying aetiology. Continued innovation in diagnostic approaches, technology, and surgical technique will be key to advancing the field and enhancing patient outcomes with CSF rhinorrhoea.

## Data Availability

No new data were created or analysed in this study. Data sharing is not applicable to this article.

## References

[1] Le C., Strong E.B., Luu Q. (2016). Management of anterior skull base cerebrospinal fluid leaks. J. Neurol. Surg. B Skull Base.

[2] Umana G.E., Pucci R., Palmisciano P., Cassoni A., Ricciardi L., Tomasi S.O., Strigari L., Scalia G., Velentini V. (2022). Cerebrospinal fluid leaks after anterior skull base trauma: A systematic review of the literature. World Neurosurg..

[3] Nelson R.F., Gantz B.J., Hansen M.R. (2015). The rising incidence of spontaneous cerebrospinal fluid leaks in the United States and the association with obesity and obstructive sleep apnea. Otol. Neurotol..

[4] Bidot S., Levy J.M., Saindane A.M., Oyesiku N.M., Newman N.J., Biousse V. (2019). Do most patients with a spontaneous cerebrospinal fluid leak have idiopathic intracranial hypertension?. J. Neuroophthalmol..

[5] Markey K.A., Mollan S.P., Jensen R.H., Sinclair A.J. (2016). Understanding idiopathic intracranial hypertension: Mechanisms, management, and future directions. Lancet Neurol..

[6] McCluskey G., Doherty-Allan R., McCarron P., Loftus A.M., McCarron L.V., Mulholland D., McVerry F., McCarron M.O. (2018). Meta-analysis and systematic review of population-based epidemiological studies in idiopathic intracranial hypertension. Eur. J. Neurol..

[7] Alromaih S.R. (2021). Pathophysiology of skull base defects and cerebrospinal fluid leak. Saudi J. Otorhinolaryngol. Head Neck Surg..

[8] Prosser J.D., Vender J.R., Solares C.A. (2011). Traumatic cerebrospinal fluid leaks. Otolaryngol. Clin. N. Am..

[9] Zapalac J.S., Marple B.F., Schwade N.D. (2002). Skull base cerebrospinal fluid fistulas: A comprehensive diagnostic algorithm. Otolaryngol. Head Neck Surg..

[10] Georgalas C., Oostra A., Ahmed S., Castelnuovo P., Dallan I., van Furth W., Harvey R.J., Herman P., Kombogiorgas D., Locatelli D. (2021). International consensus statement: Spontaneous cerebrospinal fluid rhinorrhea. Int. Forum Allergy Rhinol..

[11] Mollan S.P., Ali F., Hassan-Smith G., Botfield H., Friedman D.I., Sinclair A.J. (2016). Evolving evidence in adult idiopathic intracranial hypertension: Pathophysiology and management. J. Neurol. Neurosurg. Psychiatry.

[12] Mollan S.P., Davies B., Silver N.C., Shaw S., Mallucci C.L., Wakerley B.R., Krishnan A., Chavda S.V., Ramalingam S., Edwards J. (2018). Idiopathic intracranial hypertension: Consensus guidelines on management. J. Neurol. Neurosurg. Psychiatry.

[13] de Macedo Filho L., Machado C.C.P., Mendes G.B.B., Santana L.M.F., Ruella M.E., Grewal S., Chaichana K.L., Hinojosa A.Q., Fermo O., Almeida J.P. (2024). Spontaneous rhinorrhea and idiopathic intracranial hypertension: A complex and challenging association. Neurol. Neurochir. Pol..

[14] Phang S.Y., Whitehouse K., Lee L., Khalil H., McArdle P., Whitfield P.C. (2016). Management of CSF leak in base of skull fractures in adults. Br. J. Neurosurg..

[15] Wang E.W., Zanation A.M., Gardner P.A., Schwartz T.H., Eloy J.A., Adappa N.D., Bettag M., Bleier B.S., Cappabianca P., Carrau R.L. (2019). ICAR: Endoscopic skull-base surgery. Int. Forum Allergy Rhinol..

[16] Mostafa B.E., Khafagi A. (2004). Combined HRCT and MRI in the detection of CSF rhinorrhea. Skull Base.

[17] Jolly K., Gupta K.K., Banota A., Ahmed S.K. (2021). The effectiveness and safety of intrathecal fluorescein in the management of cerebrospinal fluid leaks. Am. J. Rhinol. Allergy.

[18] Butros S.R., Goncalves L.F., Thompson D., Agarwal A., Lee H.K. (2012). Imaging features of idiopathic intracranial hypertension, including a new finding: Widening of the foramen ovale. Acta Radiol..

[19] Morris P.P., Black D.F., Port J., Campeau N. (2017). Transverse sinus stenosis is the most sensitive MR imaging correlate of idiopathic intracranial hypertension. Am. J. Neuroradiol..

[20] Friedman D.I., Liu G.T., Digre K.B. (2013). Revised diagnostic criteria for the pseudotumor cerebri syndrome in adults and children. Neurology.

[21] Rimmer J., Belk C., Lund V.J., Swift A., White P. (2014). Immunisations and antibiotics in patients with anterior skull base cerebrospinal fluid leaks. J. Laryngol. Otol..

[22] Gonen L., Monteiro E., Klironomos G., Alghonaim Y., Vescan A., Zadeh G., Gentili F. (2015). Endoscopic endonasal repair of spontaneous and traumatic cerebrospinal fluid rhinorrhea: A review and local experience. Neurosurg. Clin. N. Am..

[23] Ratilal B.O., Costa J., Pappamikail L., Sampaio C. (2015). Antibiotic prophylaxis for preventing meningitis in patients with basilar skull fractures. Cochrane Database Syst. Rev..

[24] Wigand M.E. (1981). Transnasal ethmoidectomy under endoscopical control. Rhinology.

[25] Psaltis A.J., Schlosser R.J., Banks C.A., Yawn J., Soler Z.M. (2012). A systematic review of the endoscopic repair of cerebrospinal fluid leaks. Otolaryngol. Head Neck Surg..

[26] Komotar R.J., Starke R.M., Raper D.M.S., Anand V.K., Schwartz T.H. (2013). Endoscopic endonasal versus open repair of anterior skull base CSF leak, meningocele, and encephalocele: A systematic review of outcomes. J. Neurol. Surg. A Cent. Eur. Neurosurg..

[27] Soneru C.P., Riley C.A., Tabaee A., Kacker A., Anand V.K., Schwartz T.H. (2019). The challenge of skull base closure: Methods for reducing postoperative cerebrospinal fluid leak. World Neurosurg..

[28] Hadad G., Bassagasteguy L., Carrau R.L., Mataza J.C., Kassam A., Snyderman C.H., Mintz A. (2006). A novel reconstructive technique after endoscopic expanded endonasal approaches: Vascular pedicle nasoseptal flap. Laryngoscope.

[29] Oakley G.M., Orlandi R.R., Woodworth B.A., Batra P.S., Alt J.A. (2016). Management of cerebrospinal fluid rhinorrhea: An evidence-based review with recommendations. Int. Forum Allergy Rhinol..

[30] Wormald P.J., McDonogh M. (1997). ‘Bath-plug’ technique for the endoscopic management of cerebrospinal fluid leaks. J. Laryngol. Otol..

[31] Fiore G., Bertani G.A., Carrabba G.G., Guastella C., Marfia G., Tariciotti L., Gribaudi G.L., Mantovani G., Cristofori A.D., Locatelli M. (2021). The “Parachute” technique for the endoscopic repair of high-flow anterior skull-base CSF leaks. World Neurosurg..

[32] Leng L.Z., Brown S., Anand V.K., Schwartz T.H. (2008). “Gasket-Seal” watertight closure in minimal-access endoscopic cranial base surgery. Neurosurgery.

[33] Hosemann W., Goede U., Sauer M. (1999). Wound healing of mucosal autografts for frontal cerebrospinal fluid leaks—Clinical and experimental investigations. Rhinology.

[34] Ajlan A., Basindwah S., Hawsawi A., Omar M.A., Alsaleh S., Alrasheed A., Alroqi A., Alqurashi A. (2023). Early postoperative computed tomography scan air distribution predicts postoperative CSF leak in endoscopic skull base surgery. World Neurosurg..

[35] Souza M.N.P., Costa B.d.A.L., Santos F.R.D.R., Fortini I. (2022). Update on idiopathic intracranial hypertension management. Arq. Neuro-Psiquiatr..

[36] Abbott S., Chan F., Tahrani A.A., Wong S.H., Campbell F.E.J., Parmar C., Pournaras D.J., Denton A., Sinclair A.J., Mollan S.P. (2023). Weight management interventions for adults with idiopathic intracranial hypertension. Neurology.

[37] Piper R.J., Kalyvas A.V., Young A.M.H., Hughes M.A., Jamjoom A.A.B., Fouyas I.P. (2015). Interventions for idiopathic intracranial hypertension. Cochrane Database Syst. Rev..

[38] Zhou C., Zhou Y., Liu L., Jiang H., Wei H., Zhou C., Ji X. (2024). Progress and recognition of idiopathic intracranial hypertension: A narrative review. CNS Neurosci. Ther..

[39] Habenbacher M., Sebastnik D., Moser U., Adrianakis A., Kiss P., Alsukayt M., Pock J., Walla K., Maitz E., Tomazic P.V. (2024). The value of beta trace protein in CSF-leakage detection confirmed by endoscopic fluorescein evaluation. Rhinology.

[40] Riley C.A., Soneru C.P., Tabaee A., Kacker A., Anand V.K., Schwartz T.H. (2019). Technological and ideological innovations in endoscopic skull base surgery. World Neurosurg..

